# 3-Fluoro-*N*-(3-fluoro­benzo­yl)-*N*-(2-pyrid­yl)benzamide

**DOI:** 10.1107/S1600536808041093

**Published:** 2008-12-13

**Authors:** John F. Gallagher, Katie Donnelly, Alan J. Lough

**Affiliations:** a School of Chemical Sciences, Dublin City University, Dublin 9, Ireland; b Department of Chemistry, 80 St George Street, University of Toronto, Toronto, Ontario, Canada M5S 3H6

## Abstract

The title compound, C_19_H_12_F_2_N_2_O_2_, a 2:1 product of the reaction of 3-fluoro­benzoyl­chloride and 2-amino­pyridine crystallizes with a disordered 3-fluoro­benzene ring adopting two conformations [ratio of occupancies 0.959 (4):0.041 (4)]. In the crystal structure, there are no classical hydrogen bonds and inter­actions comprise C—H⋯O in the form 2(C—H)⋯O=C [with motif *R*_2_^1^(5)]; C—H⋯π(arene) inter­actions are also present.

## Related literature

For background information, see: Donnelly *et al.* (2008 ▶); Gallagher *et al.* (2008 ▶); McMahon *et al.* (2008 ▶); Moody *et al.* (1998 ▶). For a description of the Cambridge Structural Database, see: Allen (2002 ▶). For the parent compound, 2-(dibenzoyl­amino)pyridine, see: Weng *et al.* (2006 ▶). For related structures, see: Usman *et al.* (2002*a* ▶,*b* ▶).
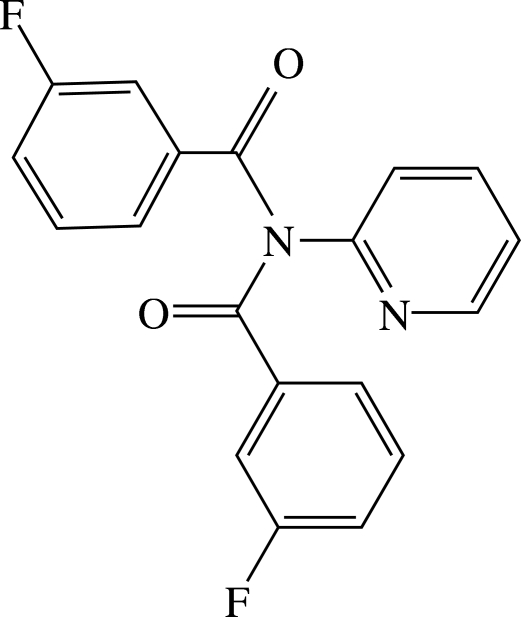


## Experimental

### 

#### Crystal data

C_19_H_12_F_2_N_2_O_2_
*M* *_r_* = 338.31
Triclinic, 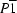

*a* = 5.4932 (4) Å
*b* = 8.1549 (5) Å
*c* = 17.9205 (15) Å
α = 78.081 (4)°
β = 89.588 (3)°
γ = 76.693 (3)°
*V* = 763.69 (10) Å^3^
*Z* = 2
Mo *K*α radiation
μ = 0.11 mm^−1^
*T* = 150 (1) K
0.34 × 0.30 × 0.12 mm

#### Data collection

Nonius KappaCCD diffractometer
Absorption correction: multi-scan (*SORTAV*; Blessing, 1995 ▶) *T*_min_ = 0.873, *T*_max_ = 0.992
5197 measured reflections
3422 independent reflections
1966 reflections with *I* > 2σ(*I*)
*R*_int_ = 0.043

#### Refinement

*R*[*F* ^2^ > 2σ(*F* ^2^)] = 0.057
*wR*(*F* ^2^) = 0.167
*S* = 1.04
3422 reflections
236 parameters
5 restraints
H-atom parameters constrained
Δρ_max_ = 0.26 e Å^−3^
Δρ_min_ = −0.32 e Å^−3^

### 

Data collection: *KappaCCD Server Software* (Nonius, 1997 ▶); cell refinement: *DENZO-SMN* (Otwinowski & Minor, 1997 ▶); data reduction: *DENZO-SMN*; program(s) used to solve structure: *SHELXS97* (Sheldrick, 2008 ▶); program(s) used to refine structure: *SHELXL97* (Sheldrick, 2008 ▶) and *SORTX* (McArdle, 1995 ▶); molecular graphics: *PLATON* (Spek, 2003 ▶); software used to prepare material for publication: *SHELXL97* and *PREP8* (Ferguson, 1998 ▶).

## Supplementary Material

Crystal structure: contains datablocks global, I. DOI: 10.1107/S1600536808041093/tk2338sup1.cif

Structure factors: contains datablocks I. DOI: 10.1107/S1600536808041093/tk2338Isup2.hkl

Additional supplementary materials:  crystallographic information; 3D view; checkCIF report

## Figures and Tables

**Table 1:** Hydrogen-bond geometry (Å, °)

*D*—H⋯*A*	*D*—H	H⋯*A*	*D*⋯*A*	*D*—H⋯*A*
C24—H24⋯O2^i^	0.95	2.53	3.097 (3)	119
C25—H25⋯O2^i^	0.95	2.46	3.063 (3)	121
C25—H25⋯*Cg*1^i^	0.95	2.79	3.606 (3)	145

Symmetry code: (i) 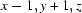. *Cg*1 is the centroid of the C11–C16 benzene ring.

**Fig. 1.**
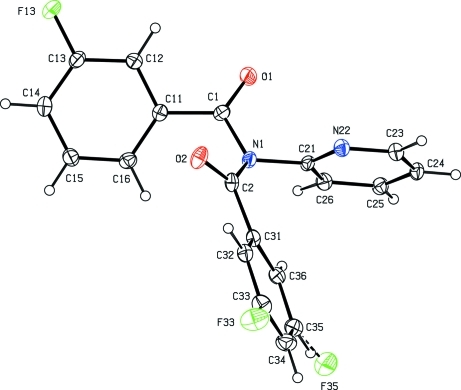
A view of (I) with the atomic numbering scheme. Displacement ellipsoids are drawn at the 30% probability level. The disordered F33/F35 sites are depicted for clarity.

**Fig. 2.**
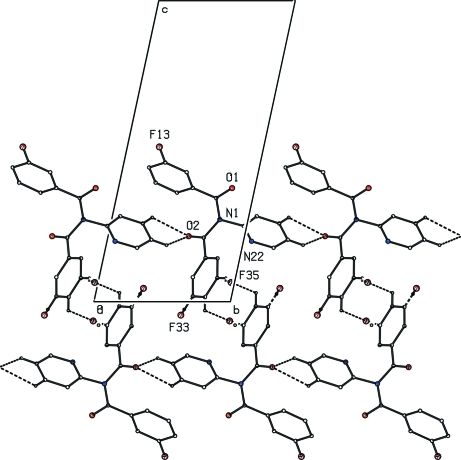
A view of the C—H···O interactions in the crystal structure of (I).

**Fig. 3.**
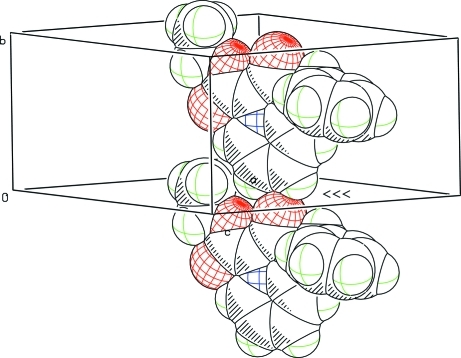
A view of the 2x(C—H)···O=C interaction in POZWUW crystal structure with atoms drawn as their van der Waals spheres (Moody et al., 1998).

**Fig. 4.**
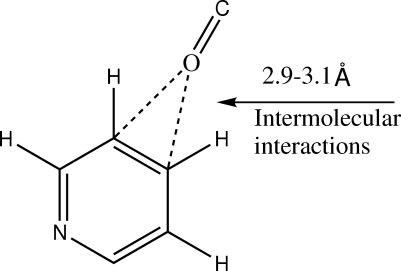
The CSD instructions and search criteria for the 2x(C—H)···O=C interaction in related structures.

## supplementary crystallographic information

### Comment

Our group is completing a structural systematic study of fluoro-*N*'-(pyridyl)benzamide isomers (Donnelly *et al.*, 2008) and we are adding to our research with the analogous difluoro-*N*-(pyridyl)benzamide series (McMahon *et al.*, 2008) (Scheme 1).

In the chemical synthesis of either the mono- or di-fluoro derivatives and when using the *ortho*-aminopyridine, two products can be isolated as either the 1:1 or 2:1 benzoyl:pyridine components, and with yields and ratios depending on the reaction conditions. We have reported the structure of the 1:1 derivative, 2,3-difluoro-*N*-(2-pyridyl)benzamide (Gallagher *et al.*, 2008), and now report a 2:1 relative of this compound, namely 3-fluoro-*N*'-(3-fluorobenzoyl)-*N*'-(2-pyridinyl)benzamide (I) (Figs 1 & 2). The parent compound 2-(dibenzoylamino)pyridine has been reported previously (Weng *et al.*, 2006) as well as the compounds *N*,*N*-dibenzoyl-4-chloroaniline and 4-acetyl-*N*,*N*-dibenzoylphenylamine (Usman *et al.*, 2002*a*,b).

In the crystal structure of (I), there are no classical hydrogen bonds and the weaker interactions present consist of C—H···O and C—H···π(arene) contacts. An unusual (phenyl)C—H···C=O interaction arises between neighbouring molecules as (C24—H24/C25—H25)···O2=C2^i^ [graph set *R*_2_^1^(5)] with O···C distances of 3.062 (3) and 3.097 (3) Å (symmetry code: i = *x* - 1, *y* + 1, *z*), Table 1.

A search of the literature (Allen, 2002) reveals a structure exhibiting a comparable example of hydrogen bonding and is archived in the CSD (as XOXRIL). However, in this structure the interacting molecules are offset with respect to the C=O···C_2_ moiety in the aromatic C_5_N ring. A related search yielded POZWUW (Fig. 3) (Moody *et al.*, 1998) and RINXUI which both have relatively symmetrical C=O···C_2_ distances similar to (I) and form chains along the *b* axis. In the POZWUW structure the C3/C4···O1^ii^ distances are 3.013 (3) and 3.090 (3) Å, and similar to that in (I) (symmetry code: ii = *x*, *y* - 1, *z*) (Fig. 3).

A related search for C=O···C_2_ [in C_6_] yielded 6 compounds in the same range of C···O from 2.0–3.0 Å but most were disordered, with high *R*-factors and typically had the solvent benzene as the acceptor; these are listed as BARJUZ10, LAYDAQ, MERRIK, OGOPUV, SEDLET, XICFEV (Allen, 2002).

### Experimental

Compound (I) was synthesized *via* standard condensation procedures and similar to the related syntheses reported previously (Donnelly *et al.*, 2008; McMahon *et al.*, 2008). Separation of the 1:1 and 2:1 derivatives was undertaken by using flash chromatography. Typical organic workup and washing gave the product (I) in modest yield of 25–35% as a 2:1 component of the mixture. Crystals suitable for X-ray diffraction were grown from CHCl_3_ as colourless blocks over a period of 1–2 weeks and gave a melting point of 401–406 K. The compounds gave clean ^1^H and ^13^C NMR spectra in CDC_3_ and infrared spectra (in CHCl_3_ solution, and as KBr disks).

For (I), m.p. 401–406 K (uncorrected). IR (ν_C=O_ cm^-1^): 1697(*s, br*), (CHCl_3_); 1695(*s*) (KBr).

### Refinement

Molecule (I) crystallized in the triclinic system; space group *P*1 (No. 2) assumed and confirmed by the refinement and analysis. In the final stages of refinement it was observed that there was electron density consistent with a partial occupancy F atom in a position expected for a minor orientation (site) of the F33 atom position. This new site only necessitates rotation by 180° about the C2—C31 axis in a group that is not engaged in strong hydrogen bonding.

The minor F35 site was treated initially with isotropic displacement values and in the final refinement cycles was restrained by *DFIX* values to 1.350 (5) Å, SIMU restraints of 0.2 (F33, F35) and FLAT constraints of 0.1 with the {C31···C36} benzene ring. The final refinement gave site occupancy values of 0.959 (4):0.041 (4). As the major and minor sites for the C_6_ ring are essentially coincidental it was decided to retain the major orientation with 100% occupancy for use with the restraints.

Refinement and disorder analysis: (WGHT, *R*-factor and residual electron density).

Refinement without disorder gives an *R*-factor of 0.058 WGHT = 0.0856 0.018, *R* = 0.058 and +0.40/-0.30. Refinement with F33 at variable occupancy changes site from 1.000 to 0.937. WGHT = 0.082 0.018, *R* = 0.057 and +0.39/-0.30. Final refinement and treatment of disorder gives an *R*-factor of 0.057: WGHT = 0.0816 0, *R* = 0.057 and +0.26/-0.32 [Inclusion of the minor site at F35 using *DFIX*/SIMU/FLAT restraints].

H atoms attached to C atoms were treated as riding with C—H = 0.95 Å, and with *U*_iso_(H) = 1.2*U*_eq_(C).

### Crystal data

**Table:**

C_19_H_12_F_2_N_2_O_2_	*Z* = 2
*M**_r_* = 338.31	*F*(000) = 348
Triclinic, *P*1	*D*_x_ = 1.471 Mg m^−^^3^
Hall symbol: -P 1	Melting point: 403 K
*a* = 5.4932 (4) Å	Mo *K*α radiation, λ = 0.71073 Å
*b* = 8.1549 (5) Å	Cell parameters from 2910 reflections
*c* = 17.9205 (15) Å	θ = 2.6–27.5°
α = 78.081 (4)°	µ = 0.11 mm^−^^1^
β = 89.588 (3)°	*T* = 150 K
γ = 76.693 (3)°	Block, colourless
*V* = 763.69 (10) Å^3^	0.34 × 0.30 × 0.12 mm

### Data collection

**Table:**

Nonius KappaCCD diffractometer	3422 independent reflections
Radiation source: fine-focus sealed X-ray tube	1966 reflections with *I* > 2σ(*I*)
graphite	*R*_int_ = 0.043
φ, and ω scans with κ offsets	θ_max_ = 27.5°, θ_min_ = 2.6°
Absorption correction: multi-scan (*SORTAV*; Blessing, 1995)	*h* = −7→7
*T*_min_ = 0.873, *T*_max_ = 0.992	*k* = −10→10
5197 measured reflections	*l* = −20→23

### Refinement

**Table:**

Refinement on *F*^2^	Primary atom site location: structure-invariant direct methods
Least-squares matrix: full	Secondary atom site location: difference Fourier map
*R*[*F*^2^ > 2σ(*F*^2^)] = 0.057	Hydrogen site location: inferred from neighbouring sites
*wR*(*F*^2^) = 0.167	H-atom parameters constrained
*S* = 1.04	*w* = 1/[σ^2^(*F*_o_^2^) + (0.0816*P*)^2^] where *P* = (*F*_o_^2^ + 2*F*_c_^2^)/3
3422 reflections	(Δ/σ)_max_ < 0.001
236 parameters	Δρ_max_ = 0.26 e Å^−^^3^
5 restraints	Δρ_min_ = −0.32 e Å^−^^3^

### Fractional atomic coordinates and isotropic or equivalent isotropic displacement parameters (Å^2^)

**Table:**

	*x*	*y*	*z*	*U*_iso_*/*U*_eq_	Occ. (<1)
F13	1.4634 (3)	0.23528 (17)	0.51411 (9)	0.0438 (4)	
F33	0.7405 (3)	0.6582 (2)	−0.04453 (9)	0.0534 (6)	0.959 (4)
F35	0.085 (3)	0.975 (3)	0.064 (3)	0.072 (17)	0.041 (4)
O1	1.0215 (3)	0.83326 (19)	0.37748 (10)	0.0389 (5)	
C1	0.9639 (4)	0.7323 (3)	0.34430 (14)	0.0268 (6)	
C11	1.0136 (4)	0.5439 (3)	0.37798 (13)	0.0247 (5)	
C12	1.2170 (4)	0.4755 (3)	0.42993 (14)	0.0283 (6)	
C13	1.2672 (4)	0.3023 (3)	0.46246 (14)	0.0302 (6)	
C14	1.1272 (5)	0.1930 (3)	0.44586 (14)	0.0316 (6)	
C15	0.9220 (5)	0.2637 (3)	0.39557 (14)	0.0310 (6)	
C16	0.8640 (4)	0.4374 (3)	0.36223 (14)	0.0285 (6)	
N1	0.8296 (4)	0.7932 (2)	0.27278 (11)	0.0252 (5)	
C21	0.7380 (4)	0.9785 (3)	0.25150 (13)	0.0232 (5)	
N22	0.8718 (4)	1.0596 (2)	0.20067 (11)	0.0282 (5)	
C23	0.7874 (4)	1.2313 (3)	0.17910 (14)	0.0293 (6)	
C24	0.5739 (4)	1.3220 (3)	0.20586 (14)	0.0288 (6)	
C25	0.4409 (4)	1.2343 (3)	0.25976 (14)	0.0282 (6)	
C26	0.5261 (4)	1.0580 (3)	0.28380 (14)	0.0279 (6)	
O2	1.0823 (3)	0.5907 (2)	0.21749 (10)	0.0357 (5)	
C2	0.8926 (4)	0.7035 (3)	0.21272 (13)	0.0259 (5)	
C31	0.7138 (4)	0.7490 (3)	0.14525 (13)	0.0256 (5)	
C32	0.8076 (5)	0.6859 (3)	0.08106 (14)	0.0287 (6)	
C33	0.6501 (5)	0.7166 (3)	0.01795 (15)	0.0361 (6)	
C34	0.4039 (5)	0.8066 (3)	0.01530 (16)	0.0381 (7)	
C35	0.3124 (5)	0.8666 (3)	0.07875 (15)	0.0345 (6)	
C36	0.4653 (4)	0.8371 (3)	0.14401 (14)	0.0279 (6)	
H12	1.3185	0.5466	0.4426	0.034*	
H14	1.1701	0.0733	0.4682	0.038*	
H15	0.8200	0.1921	0.3838	0.037*	
H16	0.7212	0.4844	0.3284	0.034*	
H23	0.8801	1.2933	0.1435	0.035*	
H24	0.5183	1.4429	0.1877	0.035*	
H25	0.2943	1.2942	0.2797	0.034*	
H26	0.4418	0.9937	0.3213	0.033*	
H32	0.9764	0.6232	0.0812	0.034*	
H33	0.7134	0.6742	−0.0256	0.043*	0.041 (4)
H34	0.2998	0.8267	−0.0293	0.046*	
H35	0.1431	0.9288	0.0780	0.041*	0.959 (4)
H36	0.3993	0.8775	0.1878	0.034*	

### Atomic displacement parameters (Å^2^)

**Table:**

	*U*^11^	*U*^22^	*U*^33^	*U*^12^	*U*^13^	*U*^23^
F13	0.0402 (9)	0.0387 (8)	0.0439 (10)	−0.0050 (7)	−0.0157 (8)	0.0068 (7)
F33	0.0543 (12)	0.0759 (13)	0.0333 (11)	−0.0060 (9)	0.0036 (8)	−0.0295 (9)
F35	0.07 (3)	0.06 (3)	0.07 (4)	−0.02 (3)	−0.02 (3)	0.01 (2)
O1	0.0534 (12)	0.0265 (9)	0.0372 (11)	−0.0090 (9)	−0.0134 (9)	−0.0078 (8)
C1	0.0250 (13)	0.0276 (12)	0.0256 (14)	−0.0026 (11)	−0.0028 (10)	−0.0049 (11)
C11	0.0268 (13)	0.0234 (11)	0.0226 (13)	−0.0030 (10)	0.0023 (10)	−0.0054 (10)
C12	0.0295 (13)	0.0268 (12)	0.0292 (14)	−0.0068 (11)	−0.0019 (11)	−0.0074 (11)
C13	0.0266 (13)	0.0286 (13)	0.0302 (15)	−0.0014 (10)	−0.0038 (11)	0.0000 (11)
C14	0.0370 (15)	0.0238 (12)	0.0309 (15)	−0.0054 (11)	0.0038 (12)	−0.0008 (11)
C15	0.0361 (14)	0.0275 (13)	0.0309 (15)	−0.0112 (11)	0.0012 (12)	−0.0052 (11)
C16	0.0291 (13)	0.0313 (13)	0.0248 (14)	−0.0048 (11)	−0.0019 (11)	−0.0072 (11)
N1	0.0300 (11)	0.0192 (9)	0.0245 (11)	−0.0015 (8)	−0.0025 (9)	−0.0045 (8)
C21	0.0257 (13)	0.0181 (11)	0.0240 (13)	−0.0001 (10)	−0.0039 (10)	−0.0056 (9)
N22	0.0295 (11)	0.0240 (10)	0.0310 (12)	−0.0053 (9)	0.0021 (9)	−0.0064 (9)
C23	0.0322 (14)	0.0247 (12)	0.0308 (15)	−0.0083 (11)	0.0012 (11)	−0.0033 (11)
C24	0.0338 (14)	0.0187 (11)	0.0318 (15)	−0.0026 (10)	−0.0039 (11)	−0.0046 (10)
C25	0.0304 (13)	0.0245 (12)	0.0292 (14)	−0.0027 (10)	−0.0001 (11)	−0.0086 (10)
C26	0.0302 (13)	0.0271 (12)	0.0267 (14)	−0.0074 (11)	0.0031 (11)	−0.0057 (10)
O2	0.0382 (10)	0.0295 (9)	0.0310 (11)	0.0069 (8)	0.0042 (8)	−0.0038 (8)
C2	0.0322 (14)	0.0182 (11)	0.0261 (14)	−0.0032 (10)	0.0054 (11)	−0.0050 (10)
C31	0.0322 (13)	0.0203 (11)	0.0250 (14)	−0.0082 (10)	0.0020 (11)	−0.0044 (10)
C32	0.0302 (13)	0.0240 (12)	0.0318 (15)	−0.0063 (11)	0.0036 (11)	−0.0058 (11)
C33	0.0456 (17)	0.0386 (14)	0.0284 (15)	−0.0139 (13)	0.0076 (13)	−0.0127 (12)
C34	0.0413 (16)	0.0445 (15)	0.0321 (16)	−0.0144 (13)	−0.0033 (13)	−0.0111 (13)
C35	0.0316 (14)	0.0329 (14)	0.0400 (17)	−0.0085 (12)	−0.0006 (12)	−0.0090 (12)
C36	0.0306 (14)	0.0257 (12)	0.0299 (15)	−0.0082 (11)	0.0024 (11)	−0.0092 (11)

### Geometric parameters (Å, °)

**Table:**

F13—C13	1.361 (3)	C25—C26	1.381 (3)
F33—C33	1.353 (3)	C31—C32	1.402 (3)
F35—C35	1.347 (5)	C31—C36	1.387 (3)
O1—C1	1.207 (2)	C32—C33	1.374 (3)
C1—N1	1.420 (3)	C33—C34	1.378 (4)
O2—C2	1.211 (3)	C34—C35	1.376 (4)
C2—C31	1.493 (3)	C35—C36	1.391 (3)
N1—C2	1.420 (3)	C12—H12	0.9500
N1—C21	1.448 (3)	C14—H14	0.9500
C1—C11	1.492 (3)	C15—H15	0.9500
C11—C12	1.393 (3)	C16—H16	0.9500
C11—C16	1.394 (3)	C23—H23	0.9500
C12—C13	1.377 (3)	C24—H24	0.9500
C13—C14	1.379 (3)	C25—H25	0.9500
C14—C15	1.384 (3)	C26—H26	0.9500
C15—C16	1.382 (3)	C32—H32	0.9500
C21—N22	1.331 (3)	C33—H33	0.9500
C21—C26	1.382 (3)	C34—H34	0.9500
N22—C23	1.344 (3)	C35—H35	0.9500
C23—C24	1.375 (3)	C36—H36	0.9500
C24—C25	1.387 (3)		
			
O1—C1—N1	119.86 (19)	C32—C33—C34	122.6 (2)
O1—C1—C11	122.3 (2)	C35—C34—C33	118.5 (2)
N1—C1—C11	117.67 (18)	C34—C35—C36	120.7 (2)
C12—C11—C16	119.5 (2)	C31—C36—C35	120.1 (2)
C12—C11—C1	116.76 (18)	C13—C12—H12	120.9
C16—C11—C1	123.7 (2)	C11—C12—H12	120.9
C13—C12—C11	118.3 (2)	C13—C14—H14	121.1
F13—C13—C12	118.5 (2)	C15—C14—H14	121.1
F13—C13—C14	118.2 (2)	C16—C15—H15	119.6
C12—C13—C14	123.3 (2)	C14—C15—H15	119.6
C13—C14—C15	117.7 (2)	C15—C16—H16	119.8
C16—C15—C14	120.7 (2)	C11—C16—H16	119.8
C15—C16—C11	120.4 (2)	N22—C23—H23	118.3
C1—N1—C2	120.05 (18)	C24—C23—H23	118.3
C1—N1—C21	114.85 (17)	C23—C24—H24	120.5
C2—N1—C21	117.18 (18)	C25—C24—H24	120.5
N22—C21—C26	124.97 (19)	C26—C25—H25	120.7
N22—C21—N1	115.2 (2)	C24—C25—H25	120.7
C26—C21—N1	119.8 (2)	C25—C26—H26	121.1
C21—N22—C23	116.2 (2)	C21—C26—H26	121.1
N22—C23—C24	123.4 (2)	C33—C32—H32	120.7
C23—C24—C25	119.0 (2)	C31—C32—H32	120.7
C26—C25—C24	118.6 (2)	C32—C33—H33	118.7
C25—C26—C21	117.7 (2)	C34—C33—H33	118.7
O2—C2—N1	120.7 (2)	C35—C34—H34	120.8
O2—C2—C31	121.5 (2)	C33—C34—H34	120.8
N1—C2—C31	117.8 (2)	C34—C35—H35	119.7
C36—C31—C32	119.5 (2)	C36—C35—H35	119.7
C36—C31—C2	124.9 (2)	C31—C36—H36	119.9
C32—C31—C2	115.4 (2)	C35—C36—H36	119.9
C33—C32—C31	118.6 (2)		
			
O1—C1—C11—C12	−27.9 (3)	N1—C21—N22—C23	178.84 (18)
N1—C1—C11—C12	156.2 (2)	C21—N22—C23—C24	−1.0 (3)
O1—C1—C11—C16	149.8 (2)	N22—C23—C24—C25	2.1 (3)
N1—C1—C11—C16	−26.1 (3)	C23—C24—C25—C26	−1.0 (3)
C16—C11—C12—C13	1.8 (4)	C24—C25—C26—C21	−1.1 (3)
C1—C11—C12—C13	179.6 (2)	N22—C21—C26—C25	2.3 (3)
C11—C12—C13—F13	−179.0 (2)	N1—C21—C26—C25	−177.79 (18)
C11—C12—C13—C14	0.4 (4)	C1—N1—C2—O2	−11.0 (3)
F13—C13—C14—C15	177.5 (2)	C21—N1—C2—O2	136.5 (2)
C12—C13—C14—C15	−2.0 (4)	C1—N1—C2—C31	167.05 (19)
C13—C14—C15—C16	1.3 (4)	C21—N1—C2—C31	−45.5 (3)
C14—C15—C16—C11	0.9 (4)	O2—C2—C31—C36	160.4 (2)
C12—C11—C16—C15	−2.5 (4)	N1—C2—C31—C36	−17.6 (3)
C1—C11—C16—C15	179.9 (2)	O2—C2—C31—C32	−15.4 (3)
O1—C1—N1—C2	138.7 (2)	N1—C2—C31—C32	166.55 (19)
C11—C1—N1—C2	−45.3 (3)	C36—C31—C32—C33	1.3 (3)
O1—C1—N1—C21	−9.4 (3)	C2—C31—C32—C33	177.3 (2)
C11—C1—N1—C21	166.5 (2)	C31—C32—C33—C34	−0.1 (3)
C1—N1—C21—N22	101.8 (2)	C32—C33—C34—C35	−0.5 (4)
C2—N1—C21—N22	−47.3 (3)	C33—C34—C35—C36	0.1 (4)
C1—N1—C21—C26	−78.1 (3)	C32—C31—C36—C35	−1.7 (3)
C2—N1—C21—C26	132.8 (2)	C2—C31—C36—C35	−177.4 (2)
C26—C21—N22—C23	−1.3 (3)	C34—C35—C36—C31	1.1 (3)

### Hydrogen-bond geometry (Å, °)

**Table:**

*D*—H···*A*	*D*—H	H···*A*	*D*···*A*	*D*—H···*A*
C24—H24···O2^i^	0.95	2.53	3.097 (3)	119
C25—H25···O2^i^	0.95	2.46	3.063 (3)	121
C25—H25···Cg1^i^	0.95	2.79	3.606 (3)	145

Symmetry codes: (i) *x*−1, *y*+1, *z*.
